# Could COVID-19 represent a negative prognostic factor in patients with stroke?

**DOI:** 10.1017/ice.2020.146

**Published:** 2020-04-20

**Authors:** Antonio Siniscalchi, Luca Gallelli

**Affiliations:** 1 Department of Neurology and Stroke Unit, Annunziata Hospital of Cosenza, Cosenza, Italy; 2 Department of Health Science, School of Medicine, University of Catanzaro, Catanzaro, Italy; 3 Clinical Pharmacology Unit, Mater Domini University Hospital, Catanzaro, Italy

*To the Editor*—Coronavirus infectious disease 2019 (COVID-19) is a highly contagious disease that has become a worldwide pandemic. Coronaviruses (CoVs), positive-stranded RNA viruses, are known to cause respiratory or intestinal infections in humans and animals.^1^ Coronaviruses are known to affect the cardiovascular system.^2^

The SARS-CoV-2 virus uses the enzyme 2 receptor (ACE2) to gain entry into cells,^3^ and these receptors have been revealed in the neuronal and glial cells of the human brain. Thus, they may be a potential target of SARS-CoV-2, which might explain the death of olfactory cells in patients with COVID-19.^1^ CoVs can enter the central nervous system through 2 distinct pathways: retrograde neuronal diffusion or hematogenous diffusion. The spread of SARS-CoV-2 through the cribriform plaque of the ethmoid bone during an initial or subsequent infection phase can lead to brain involvement. In the systemic circulation, the presence of ACE2 receptors on both capillary and neuronal endothelial cells could be responsible for the subsequent spread and damage to the cerebral nervous system without substantial inflammation. The presence of CoVs in the cerebral nervous system has been confirmed in the cerebrospinal fluid and brain tissues of patients during autopsies.^4,5^

Several symptoms indicative of CNS involvement are present in approximately one-third of COVID-19 patients: dizziness, headache, impaired consciousness, ataxia, epilepsy, and acute cerebrovascular disease.^1^ Changes in the coagulation system (ie, D-dimer and platelet abnormalities)^2,6^ and in inflammatory biomarkers (eg, interleukin-6, C-reactive protein, and ferritin)^7^ have been reported in COVID-19 patients. In patients with stroke, the presence of COVID-19 could be a potential extrinsic factor in the genesis or worsening of stroke. Infection or high levels of proinflammatory biomarkers indicate significantly increased risk of ischemic stroke, especially in the elderly.^8-10^ The onset or worsening of a stroke in these patients could be caused either by direct damage of the CoVs on the nervous system and/or by an activation of the mechanisms of COVID-19 inflammation induced as well coagulation disorders. As the disease spreads and new evidence emerges, we need to identify the existence of additional pathophysiological mechanisms of stroke in COVID-19 patients. We should establish a prospective registry of these patients to better identify the factors most responsible for a possible greater onset or worse prognosis of stroke in these patients and to identify and/or predict a better or lesser response of these patients to thrombolytic treatments.
